# Event-Scale Responses of Phytoplankton and Heterotrophic Bacterial Biomass and Production to Super Typhoon Maria in the East China Sea

**DOI:** 10.3390/biology15131007

**Published:** 2026-06-25

**Authors:** Tzong-Yueh Chen, Nien En Thai, Chao-Chen Lai, Liang-Yu Chen, Fuh-Kwo Shiah, Gwo-Ching Gong

**Affiliations:** 1 Institute of Marine Environment and Ecology, National Taiwan Ocean University, Keelung 202301, Taiwan; 0063b046@email.ntou.edu.tw (N.E.T.); clyly1226@gmail.com (L.-Y.C.); fkshiah@gate.sinica.edu.tw (F.-K.S.); gwoching.gong@gmail.com (G.-C.G.); 2 Center of Excellence for the Oceans, National Taiwan Ocean University, Keelung 202301, Taiwan; 3 Department of Science Education, National Taipei University of Education, Taipei 106320, Taiwan; ccjosephlai@mail.ntue.edu.tw; 4 Research Center for Environmental Changes, Academia Sinica, Taipei 115201, Taiwan

**Keywords:** typhoon, chlorophyll, nutrient, heterotrophic bacteria, phytoplankton, East China Sea

## Abstract

Typhoons are major physical disturbances in marginal seas, yet their event-scale impacts on microbial processes and carbon cycling remain poorly constrained. Here, we investigated the biogeochemical responses to Super Typhoon Maria (2018) in the East China Sea using combined field observations and satellite data. While surface temperature, nutrients, and chlorophyll-a (Chl-a) showed no significant changes, depth-integrated nutrients and Chl-a increased markedly, revealing a clear decoupling between surface and depth-integrated responses driven by vertical mixing and upwelling. Satellite observations further showed that phytoplankton enhancement was short-lived, with Chl-*a* returning to background levels within one week. This rapid attenuation likely reflects transient nutrient supply and strong grazing pressure. In contrast, microbial responses were characterized by increased bacterial specific growth rate without significant changes in biomass or production, indicating enhanced microbial turnover. Together, these results suggest that typhoon forcing promotes rapid and vertically structured carbon processing through the microbial loop without increasing biomass accumulation. This highlights the importance of temporal resolution and vertical structure in understanding ecosystem responses to episodic disturbances in marginal seas.

## Simple Summary

Typhoons are often thought to enhance ocean productivity by cooling surface waters and bringing nutrients to the surface. However, our study of Super Typhoon Maria (2018) in the East China Sea shows a more complex response. Field measurements indicated little change in surface temperature, nutrients, and chlorophyll-*a*, likely reflecting the timing of sampling relative to the typhoon passage, when transient surface signals had already weakened. In contrast, depth-integrated data revealed increases in nutrients and phytoplankton biomass, indicating that typhoon effects were more persistently expressed below the surface. Satellite observations further showed that the phytoplankton increase lasted for only about one week, shorter than reported in previous studies. This short duration likely resulted from a rapid decline in nutrient supply and strong grazing pressure, which quickly removed phytoplankton biomass. For microbes, bacterial biomass and production did not increase significantly, but bacterial growth rates rose, indicating more active microbial processing. Overall, our results suggest that typhoons do not necessarily increase biomass, but instead accelerate carbon cycling by enhancing microbial activity. This highlights the importance of considering both vertical structure and timing when evaluating the ecological impacts of extreme weather events in the ocean.

## 1. Introduction

Tropical cyclones (typhoons) are among the most intense physical disturbances in the ocean and are widely recognized for their impacts on ecosystem functioning. Strong wind forcing associated with typhoons can deepen the mixed layer, enhance vertical turbulent mixing, and induce upwelling or uplift of the nutricline, thereby supplying nutrient-rich subsurface water to the euphotic zone [1,2,3,4]. Previous studies have shown that these physical processes can result in rapid sea surface cooling, enhanced nutrient availability, and increased chlorophyll-a concentrations or primary production after storm passage [5,6,7,8]. However, autotrophic and heterotrophic microbial responses to individual typhoon events are highly variable, ranging from negligible changes to pronounced chlorophyll increases and shifts in community composition, e.g., [5,9,10,11,12,13]. The connection between the magnitude of the response and the oceanic conditions prevailing under the tropical cyclone remains uncertain.

Although numerous studies have investigated typhoon-induced responses in primary productivity and phytoplankton dynamics in the open ocean, much of the existing understanding is derived from satellite-based remote sensing, e.g., [9,10,11,12]. While satellite observations provide valuable large-scale coverage, their application in coastal (Case-2) waters is inherently limited due to the influence of suspended particles and chromophoric dissolved organic matter on optical properties. These factors can introduce substantial uncertainties in chlorophyll-*a* (Chl-*a*) retrievals and may lead to misinterpretation of phytoplankton biomass [14,15,16]. Moreover, field observations during typhoon events remain scarce due to logistical and safety constraints, despite the increasing frequency and intensity of extreme weather events under climate change. Consequently, direct field measurements are essential for resolving rapid ecosystem responses and associated biogeochemical feedbacks in coastal environments.

The East China Sea (ECS) shelf, one of the largest marginal seas in the world, represents a highly dynamic biogeochemical system influenced by riverine inputs, shelf–open ocean exchange, and Kuroshio intrusion [17,18]. This region experiences frequent typhoon passages each year, making it an ideal natural laboratory for investigating ecosystem responses to episodic physical forcing. Despite this, studies addressing microbial responses in the ECS during typhoon events remain limited. Previous work has largely focused on phytoplankton dynamics or bulk productivity. For instance, Chung et al. [6] documented post-typhoon phytoplankton succession over a weekly timescale, whereas Shiah et al. [5] examined variations in primary and bacterial production based on seasonal comparisons rather than resolving changes associated with a single event. As a result, event-scale microbial responses and their role in mediating biogeochemical processes remain poorly understood.

Super Typhoon Maria, a Category 5 storm, traversed the East China Sea (ECS) shelf in July 2018 and provided a rare opportunity to examine event-scale ecosystem responses. The typhoon reached its maximum intensity on 9 July, with sustained wind speeds up to 54 m s^−1^. It subsequently passed the northern tip of Taiwan and moved northwestward toward the adjacent continental margin at a forward speed of ~30 km h^−1^. Although Maria weakened to a severe typhoon prior to landfall on the continent on 11 July (09:10 UTC+8), it remained highly energetic, with maximum sustained winds of 48 m s^−1^ and a minimum central pressure of 960 hPa, generating storm surges of ~2.5 m and significant wave heights of up to 9 m along the coastal region.

In this study, we conducted coordinated field observations before and after the passage of Typhoon Maria, complemented by satellite remote sensing, to investigate the impacts of a single typhoon event on hydrographic conditions and microbial dynamics. Measurements included physical properties, nutrient concentrations, chlorophyll-*a*, and microbial parameters. This combined approach enables direct assessment of microbial and biogeochemical responses at the event scale while providing spatial context from satellite observations, thereby offering new insights into the mechanisms linking physical forcing to ecosystem dynamics in marginal seas.

## 2. Materials and Methods

### 2.1. Study Area and Sampling

**Figure 1:**
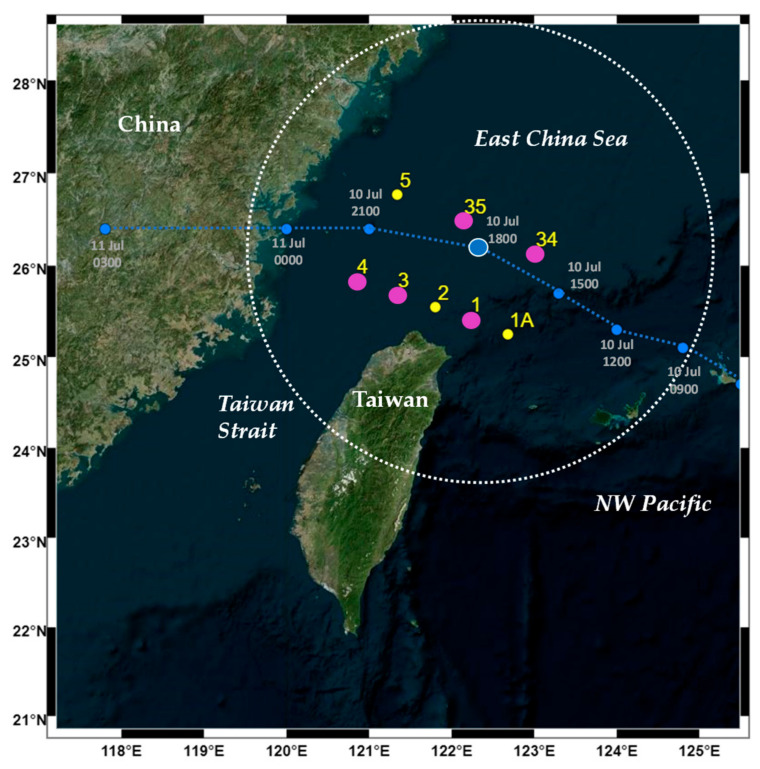
Study area showing sampling sites and the track of Super Typhoon Maria (blue deshed line) in the East China Sea. Pink dots denote stations with primary production measurements, and white circle indicates the maximum radius of gale-force winds.

The cruise was conducted aboard the R/V Ocean Researcher I in the subtropical southern East China Sea shelf, northeast of Taiwan and west of the Ryukyu Islands along the northwestern Pacific margin, from 6–9 July and 11–16 July 2018, encompassing the periods immediately before and after the passage of Super Typhoon Maria on 10 July 2018 (Figure 1; Table 1). Throughout the cruise, photosynthetically active radiation (PAR) above the sea surface was continuously monitored using an irradiance sensor (Biospherical, San Diego, CA, USA). Seawater sampling was performed at nine stations during both pre- and post-typhoon periods. At each station, seawater samples were collected from six to nine depths within the upper 200 m depth (Supplementary Table S1) using a rosette sampler fitted with 20-L Go-Flo bottles (General Oceanics, Miami, FL, USA) and an integrated CTD profiler (Seabird, Bellevue, WA, USA). Water samples collected from every sampled depth were used for all subsequent analyses. In addition to measuring temperature and salinity, vertical profiles of underwater PAR were obtained using a sensor (Chelsea Technologies, Yateley, Hamsphire, UK) mounted on the CTD. From these data, the vertical light attenuation coefficient (K_d_) was calculated. The depth of the euphotic zone (Z_e_) was defined as the depth at which surface PAR is reduced to 1%, estimated as 4.605/K_d_. The mixed layer depth (MLD) was determined based on a change in density (sigma-*t*) of 0.125 kg m^−3^ from the surface, following the criterion described by Vaulot and Marie [19].

### 2.2. Chemical Determinations

Particulate matter was operationally defined as material retained on pre-combusted GF/F filters (Whatman, Marlborough, MA, USA), representing particles larger than the nominal pore size of 0.7 µm, while dissolved constituents were defined as those passing through the same filters. Nutrient samples—including nitrate, nitrite, phosphate, and silicate—were collected in 100 mL acid-cleaned polypropylene bottles, immediately flash-frozen in liquid nitrogen, and stored at −20 °C until analysis. Nitrate was reduced to nitrite using cadmium-copper filings, and concentrations were determined via the diazo-pink colorimetric method [20]. Nitrite was measured using the same method without a reduction process. Phosphate concentrations were quantified using the molybdenum-blue method, and silicate concentrations were determined using the molybdate-blue method, both following the protocols described by Parsons et al. [20]. The nitracline depth was defined at the depth the nitrate concentration equals 1 μM [21]. Samples for chlorophyll a (Chl-a) were collected by filtering 1~2 L of seawater onto GF/F filters and stored at −20 °C until analysis. Chl-a was extracted using 100% acetone, and concentrations were measured fluorometrically according to the method of Welschmeyer [22].

### 2.3. Biological Determinations

#### 2.3.1. Primary Production: Photosynthesis-Irradiance (*P^B^*–E) Experiments

Primary production was quantified using the ^14^C assimilation method as described by Parsons et al. [20]. Briefly, surface seawater samples for photosynthesis–irradiance (*P^B^*–E) experiments were collected from five stations (see Figure 1) both before and after the passage of Typhoon Maria. *P^B^*–E curves were obtained using a surface seawater-cooled incubator equipped with artificial lighting (100 W). Each sample was amended with 10 µCi of ^14^C and incubated under ten different levels of photosynthetically active radiation (PAR): 0, 65, 130, 260, 400, 480, 800, 950, 1365, and 2000 µE m^−2^ s^−1^ (where 1 Einstein (E) = 1 mole photons). After a 2-h incubation, duplicate 5 mL subsamples were collected for both dissolved primary production (DPP) and particulate primary production (PPP). DPP was determined from the filtrate that passed through a 0.22-µm membrane filter (Millipore, Burlington, MA, USA), while PPP was collected on GF/F filters. Following acidification with HCl and the addition of scintillation cocktail (PerkinElmer, Waltham, MA, USA), both DPP and PPP were measured via liquid scintillation counting. Vertical profiles of DPP and PPP were estimated by fitting the observed *P^B^*–E relationships using models from Webb et al. [23] and Platt et al. [24]. Daily depth-integrated primary production was then calculated using the trapezoidal integration method.

#### 2.3.2. Heterotrophic Bacteria

Bacterial abundance and production were determined at all depths using flow cytometry [25] and ^3^H-thymidine incorporation [26], respectively. For abundance, samples were preserved with paraformaldehyde (1% final concentration), flash-frozen in liquid nitrogen, stored at −80 °C, stained with SYBR Green I, and analyzed using a flow cytometer (CyFlow, PARTEC, Görlitz, Germany) with internal bead standards. Bacterial production (BP) was measured in triplicate by incubating samples amended with ^3^H-thymidine at in situ temperature for 2 h. Incubations were terminated with formaldehyde, and incorporation rates were quantified by liquid scintillation counting (PerkinElmer, USA). Carbon-based estimates were derived using conversion factors of 1.18 × 10^18^ cells mol^−1^ thymidine [26] and 2 × 10^−14^ g C cell^−1^ [27]. Bacterial respiration (BR) was measured at two to three selected depths per station, representing the upper, middle, and deep water layers. The deepest sample was collected either near the bottom or at 200 m, depending on station depth. Prefiltered samples (1.2 μm) were incubated in the dark for 48 h in 300 mL BOD bottles at near in situ temperature. Dissolved oxygen was measured before and after incubation using a modified Winkler method [28], and respiration rates were converted to carbon units using a Redfield-based respiratory quotient (C:O_2_ = 106:138). Because bacterial respiration was not measured at all depths, a BP–BR regression was established from the measured data in this study and used to estimate bacterial respiration at each depth, following the conceptual framework of Robinson [29].

### 2.4. Satellite Data Acquisition

Satellite-derived chlorophyll-*a* (Chl-*a*) and sea surface temperature (SST) data were obtained from the Copernicus Marine Environment Monitoring Service (CMEMS). Level-4 (L4) global ocean color Chl-*a* data (product ID: OCEANCOLOUR_GLO_BGC_L4_MY_009_104; https://doi.org/10.48670/moi-00281), with a spatial resolution of 4 km and a long-term record since 1997, were used to examine spatial and temporal variability in phytoplankton biomass. Data were extracted for the region 118–124° E and 24–28° N during 6–16 July 2018, encompassing the period before, during, and after the passage of Typhoon Maria.

SST data were derived from the Operational Sea Surface Temperature and Ice Analysis (OSTIA) L4 reprocessed product [30] (Product ID: SST_GLO_SST_L4_REP_OBSERVATIONS_010_011; https://doi.org/10.48670/moi-00168), which provides daily, gap-free foundation SST fields at a spatial resolution of 0.05° × 0.05° by integrating satellite and in situ observations. This product was selected to minimize data gaps caused by cloud cover and to ensure spatially complete SST fields under typhoon conditions. SST data were extracted for the same domain (118–124° E, 24–28° N) and time period (6–16 July 2018). Spatial distributions were visualized using Surfer 9 (Golden Software, Golden, CO, USA), and regional mean SST values were calculated for a subdomain (120.725–123.025° E, 25.225–26.725° N) to quantify temporal changes before and after the typhoon passage.

### 2.5. Statistical Analysis

All statistical analyses were performed using SPSS 22 (IBM, Armonk, NY, USA). Linear regression was applied to examine relationships among variables. To evaluate typhoon-induced changes while accounting for spatial variability among stations, log response ratios were calculated for each parameter as ln (post-typhoon/pre-typhoon) at each station. One-sample *t*-tests (*t*-test thereafter) were then conducted to determine whether the mean log response ratio differed significantly from zero, indicating systematic increases or decreases following the typhoon. For variables containing zero or below-detection-limit values, a small constant equivalent to one-half of the detection limit was added prior to log transformation [31]. Statistical significance was defined at *p*-value < 0.05. All reported values are presented as mean ± standard deviation (SD).

## 3. Results

### 3.1. Hydrological and Environmental Parameters

Prior to the typhoon, sea surface temperature (SST; recorded from the CTD) at nine stations ranged from 26.84 to 28.24 °C, with an average of 27.76 ± 0.49 °C (Table 2). Following the typhoon’s passage, SST decreased, ranging from 22.52 to 28.16 °C, with an average of 26.96 ± 1.88 °C (Table 2). The most pronounced cooling was observed at Station 1, where SST dropped from 27.90 °C to 22.52 °C, indicating an intensification of topographic upwelling in this area. In contrast, SST at the other stations exhibited only minor reductions or remained relatively unchanged, with differences that were not statistically significant.

The concentration of dissolved inorganic nitrogen (DIN), including nitrate, nitrite, and ammonium, was examined in relation to the typhoon event. Prior to the typhoon, surface DIN concentrations ranged from 0.03 to 0.46 μM, with an average of 0.17 ± 0.13 μM (Table 2). Following the typhoon, surface DIN concentrations increased with a large variability, ranging from under the detection limit to 2.07 μM, with an average of 0.46 ± 0.66 μM (Table 2). A *t*-test revealed no statistically significant difference in surface DIN concentrations between pre- and post-typhoon periods. Integrated DIN concentrations over the water column ranged from 0.02 to 1.01 mmol m^−2^ prior to the typhoon, with an average of 0.31 ± 0.33 mmol m^−2^ (Table 2). After the typhoon, integrated DIN ranged from 0.04 to 1.14 mmol m^−2^, with an average of 0.41 ± 0.40 mmol m^−2^ (Table 2). A *t*-test indicated a statistically significant increase in integrated DIN following the typhoon (*p*-value < 0.05; Table 2), with an average increase of 45.10 ± 0.43%. These results suggest that the typhoon-induced physical disturbances enhanced vertical mixing, facilitating the upward transport of nutrients from deeper layers of the water column. Because nitrogen is generally the primary limiting nutrient in the northwestern Pacific and East China Sea shelf region, DIN was used as the main indicator of typhoon-induced nutrient supply. Phosphate and silicate data are provided in Supplementary Table S2 to support a more complete evaluation of hydrochemical responses.

Surface Chl-*a* concentrations ranged from 0.20 to 0.52 mg m^−3^ (0.34 ± 0.12 mg m^−3^) before the typhoon and increased to 0.15–1.54 mg m^−3^ (0.53 ± 0.46 mg m^−3^) afterward (Table 2), with elevated values observed at five of the eight stations. However, no significant difference was detected in surface Chl-*a* between the pre- and post-typhoon periods (*p*-value > 0.05). In contrast, depth-integrated Chl-*a* increased significantly from 33.49 ± 8.39 to 45.63 ± 14.96 mg m^−2^ (Table 2; *t*-test; *p*-value < 0.05), representing an average enhancement of 38.6 ± 33.8%. The increase in integrated Chl-*a* suggests enhanced phytoplankton growth associated with typhoon-induced nutrient enrichment.

### 3.2. Primary Production

Based on the *P^B^*–E relationship, pre-typhoon observations indicated PPP values ranging from 335 to 790 mgC m^−2^ d^−1^, with an average of 584 ± 195 mgC m^−2^ d^−1^. Following the typhoon, PPP increased to a range of 518 to 1823 mgC m^−2^ d^−1^, averaging 935 ± 543 mgC m^−2^ d^−1^. Concurrently, DPP prior to the typhoon ranged from 263 to 654 mgC m^−2^ d^−1^, with an average of 504 ± 164 mgC m^−2^ d^−1^. Post-typhoon DPP exhibited a broader range of 388 to 2827 mgC m^−2^ d^−1^, with an average of 989 ± 1040 mgC m^−2^ d^−1^. Total primary production (TPP), calculated as the sum of PPP and DPP, ranged from 598 to 1444 mgC m^−2^ d^−1^ before the typhoon, with an average of 1088 ± 357 mgC m^−2^ d^−1^, and increased to a range of 951 to 4650 mgC m^−2^ d^−1^ after the typhoon, averaging 1923 ± 1570 mgC m^−2^ d^−1^. Despite the observed increase in mean primary production following the typhoon, a *t*-test indicated that the differences were not statistically significant. The proportion of extracellular release, expressed as the percentage of extracellular release (PER), was calculated as the ratio of dissolved primary production (DPP) to total primary production (TPP). Prior to the typhoon, PER ranged from 44.0 to 48.6%, with a mean value of 46.3 ± 1.8%. Following the typhoon, PER exhibited a broader range (40.8–60.8%) and a higher variability, with a mean value of 46.3 ± 8.3%. An increase in PER was observed only at Station 1, whereas no consistent pattern was evident across the remaining stations. Statistical analysis indicated that PER did not differ significantly between the pre- and post-typhoon periods (*p*-value > 0.05; Table 3).

### 3.3. Heterotrophic Bacteria

Prior to the typhoon event, BB ranged from 493.0 to 1047.5 mgC m^−2^, with an average of 670.5 ± 175.4 mgC m^−2^. Following the typhoon, BB values varied between 354.4 and 1180.3 mgC m^−2^, averaging 634.9 ± 268.0 mg C m^−2^. An increase in BB was observed at stations 5 and 34 after the typhoon, whereas other stations showed a decrease; however, these spatial differences were not statistically significant (*t*-test, *p*-value > 0.05). Before the typhoon, the highest BB was recorded at station 2 (1047.5 mgC m^−2^), and the lowest at station 5 (493.0 mgC m^−2^). After the typhoon, the maximum BB occurred at station 34 (1180.3 mgC m^−2^), while the minimum was observed at station 1 (354.4 mgC m^−2^).

Prior to the passage of the typhoon, BP ranged from 69.6 to 114.2 mgC m^−2^ d^−1^, with an average of 95.2 ± 16.0 mgC m^−2^ d^−1^. Following the typhoon, BP displayed a broader range, from 64.6 to 183.2 mgC m^−2^ d^−1^, with an increased average of 116.4 ± 36.2 mgC m^−2^ d^−1^. A general increasing trend in BP was observed across most stations after the typhoon, except at stations 4 and 35, where BP declined. Station-specific analysis revealed that the maximum pre-typhoon BP was recorded at station 3 (114.2 mgC m^−2^ d^−1^), while the post-typhoon peak occurred at station 34 (183.2 mgC m^−2^ d^−1^). In contrast, the lowest BP values were consistently detected at station 4, with 74.1 mgC m^−2^ d^−1^ before and 64.6 mgC m^−2^ d^−1^ after the typhoon. Like bacterial biomass, BP values pre- and post-typhoon showed no statistical significance (*t*-test; *p*-value > 0.05).

The bacterial specific growth rate (Bμ) was calculated by dividing BP by BB. Prior to the typhoon, the depth-integrated average Bμ ranged from 0.10 to 0.21 d^−1^, with an average of 0.15 ± 0.03 d^−1^. Following the typhoon event, Bμ increased to a range of 0.12 to 0.31 d^−1^, with an average of 0.20 ± 0.07 d^−1^. A *t*-test revealed a statistically significant post-typhoon increase in Bμ (*p*-value < 0.05; Table 3), with an average relative elevation of 35.0 ± 0.4%. Before the typhoon, the highest Bμ was recorded at station 3 (0.21 d^−1^) and the lowest at station 2 (0.10 d^−1^). After the typhoon, the maximum Bμ was observed at station 1 (0.31 d^−1^), while the minimum was found at station 4 (0.12 d^−1^).

This study included direct measurements of bacterial respiration (BR) at two to three depths per station. Because bacterial respiration was not measured at all depths, depth-integrated BR was estimated from the empirical BP–BR relationship established using the measured data from the present study, following the conceptual framework of Robinson [29]. The resulting regression was:

BR = (1.16 ± 0.18) × BP^(0.61±0.16)^ (R^2^ = 0.41; *p*-value < 0.01; *n* = 22).

Before the passage of the typhoon, modeled BR values ranged from 2494 to 4371 mgC m^−2^ d^−1^, with a mean of 3388 ± 784 mgC m^−2^ d^−1^. Following the typhoon, BR values ranged from 2488 to 5020 mgC m^−2^ d^−1^, with an average of 3833 ± 876 mgC m^−2^ d^−1^. An increase in averaged BR after the typhoon event was observed. However, there was no significant difference in BR between pre- and post-typhoon periods.

## 4. Discussion

### 4.1. Surface Cooling and Subsurface Nutrient Enhancement

Typhoon-induced cooling and nutrient enrichment in the upper ocean are commonly attributed to enhanced vertical mixing and upwelling, which entrain nutrient-rich subsurface waters into the euphotic zone [2,32,33], thereby stimulating primary production [5,10,11]. However, the present study revealed a decoupled response between surface and water-column properties. In situ observations showed no significant change in surface temperature or dissolved inorganic nitrogen (DIN) between pre- and post-typhoon conditions (Table 2). This apparent lack of response is likely related to the timing of sampling, which was conducted 3–6 days (Table 1) after the typhoon passage, when transient surface signals induced by storm forcing may have already partially recovered.

**Figure 2:**
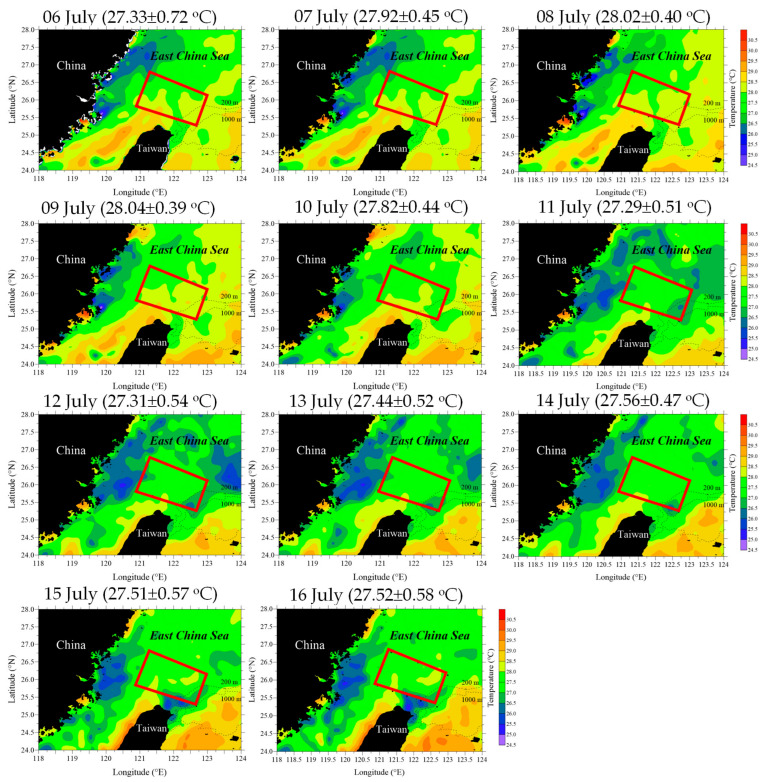
Temporal variation in satellite-derived sea surface temperature (SST) from 6 to 16 July 2018. The red box delineates the study area, and values in parentheses indicate the mean SST ± standard deviation calculated within the region.

Satellite-derived sea surface temperature (SST) from CMEMS provides independent evidence that typhoon-induced cooling did occur (Figure 2). Averaged over the study region (120.725–123.025° E, 25.225–26.725° N), SST remained relatively stable prior to the typhoon (27.73–28.03 °C during 6–9 July 2018). Following the typhoon, SST declined to 27.29–27.56 °C during 11–16 July, corresponding to an overall cooling of ~0.5 °C relative to pre-typhoon conditions. This magnitude of cooling is consistent with previous studies reporting typhoon-induced SST decreases, e.g., [15,34,35], and long-term analyses indicating typical reductions of ~0.42 ± 0.02 °C [36]. The discrepancy between satellite and in situ observations therefore reflects the rapid temporal evolution of surface responses, which may dissipate within a few days following the relaxation of wind forcing.

A similar pattern was observed for nutrient dynamics. Surface DIN concentrations showed no significant difference between pre- and post-typhoon periods, suggesting that the initial nutrient enrichment at the surface had weakened by the time of sampling (Table 2). In contrast, water column-integrated DIN increased significantly after the typhoon, indicating a sustained enhancement of nutrient supply at depth (Table 2). This vertical decoupling implies that typhoon-induced processes primarily affected subsurface layers, where the nutrient signal persisted longer than at the surface. Riverine inputs are unlikely to account for this increase, as their influence is generally confined to nearshore surface waters. Instead, the observed increase in integrated DIN is consistent with upwelling-driven nutrient entrainment.

Supporting this interpretation, the mixed layer depth (defined by a 0.125 kg m^−3^ density difference from the surface) became significantly shallower at six out of eight stations after the typhoon, while both the nitracline depth and the depth of maximum buoyancy frequency shoaled, indicating reduced water column stability and upward displacement of nutrient-rich waters. Such vertical restructuring is characteristic of typhoon-induced upwelling [33] and is further supported by the observed temperature profiles. In the ECS, these processes may be further enhanced by the shoreward intrusion of the Kuroshio, which can facilitate the upwelling of nutrient-rich subsurface waters [37].

Together, these results suggest that Typhoon Maria induced a transient surface response but a more persistent subsurface signal. The rapid recovery of surface temperature and nutrient concentrations, contrasted with sustained increases in depth-integrated nutrients, highlights the importance of sampling timing and vertical structure in interpreting typhoon impacts. This finding underscores that surface observations alone may underestimate the magnitude of typhoon-induced biogeochemical perturbations in marginal seas.

### 4.2. Decoupled and Transient Chlorophyll Responses by Field and Satellite Observations

The responses of Chl-a to Typhoon Maria exhibited a pattern consistent with that observed for nutrient dynamics, characterized by a clear decoupling between surface and depth-integrated signals. As discussed above, surface DIN did not differ significantly between pre- and post-typhoon conditions, whereas water depth-integrated DIN increased markedly, indicating enhanced nutrient supply through typhoon-induced mixing and upwelling. A similar pattern was observed for Chl-a: while surface concentrations showed no significant change, depth-integrated Chl-a increased significantly after the typhoon (Table 2; *p* < 0.05). This apparent inconsistency between surface and integrated Chl-a signals suggests that phytoplankton biomass increased shortly after the typhoon due to enhanced nutrient supply, but that the signal at the surface had already diminished by the time of sampling. Satellite- and model-based studies have shown that typhoon-induced phytoplankton blooms typically peak within 2–4 days after storm passage [8], followed by a decline driven by sinking and grazing processes. Given that post-typhoon sampling in this study was conducted 3–6 days after passage (Table 1), it is likely that the surface Chl-a signal had already begun to dissipate. Meanwhile, the increase in depth-integrated Chl-a suggests that a portion of the newly produced phytoplankton biomass had been redistributed to deeper layers through sinking, resulting in an apparent enhancement of water column biomass without a corresponding surface signal.

**Figure 3:**
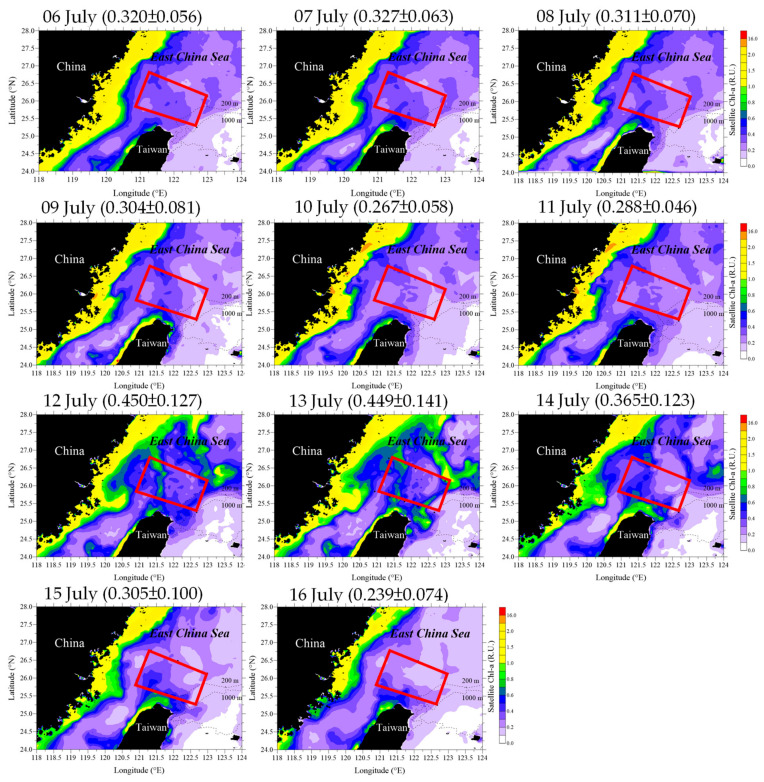
Temporal variation in satellite-derived chlorophyll-a concentrations expressed in relative units (R.U.) from 6 to 16 July 2018. The red box delineates the study area, and values in parentheses indicate the mean Chl-a ± standard deviation calculated within the region.

**Figure 4:**
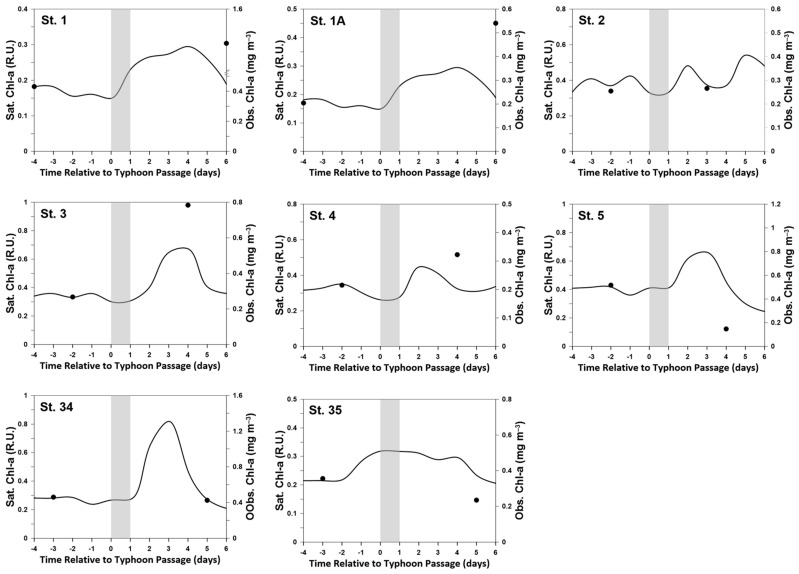
Satellite-derived chlorophyll-*a* time series at each station (St.) relative to typhoon passage. Solid lines represent chlorophyll-*a* in relative unit (R.U.), and black dots indicate *in situ* measurements. The grey shaded area denotes the typhoon period.

Satellite observations provide further support for this interpretation. CMEMS-derived Chl-a was expressed in relative units (R.U.) to emphasize temporal and spatial variations rather than absolute concentration differences. The results further supported the transient and spatially heterogeneous nature of the phytoplankton response to Typhoon Maria (Figure 3). Regional mean Chl-a remained relatively low before the typhoon, ranging from 0.304 to 0.327 R.U. during 6–9 July, and decreased to 0.267 R.U. on the typhoon passage day. After the typhoon, Chl-a rapidly increased to 0.450–0.449 R.U. on 12–13 July, but then declined to 0.239 R.U. by 16 July. This pattern indicates that the surface phytoplankton enhancement was short-lived and spatially patchy, consistent with a brief nutrient pulse followed by rapid attenuation through biomass redistribution and biological removal processes. Satellite-derived Chl-a data revealed transient increases in phytoplankton biomass at all stations following the typhoon, although the timing of peak concentrations varied spatially (Figure 4). Most stations exhibited peak Chl-a within 2–4 days after the typhoon, consistent with previous modeling and observational studies [12]. In several cases, the timing of field sampling did not coincide with the satellite-derived peak, leading to an underestimation of the surface response. This temporal mismatch highlights the limitations of discrete in situ sampling in capturing rapid and short-lived phytoplankton dynamics associated with typhoon forcing.

However, the comparison between in situ and satellite-derived Chl-a revealed a weak correlation (r^2^ = 0.094), indicating substantial uncertainties in satellite retrievals over the continental shelf. Such discrepancies are expected in optically complex (Case-2) waters, where suspended particles and chromophoric dissolved organic matter can interfere with ocean color signals [37,38]. In addition, diel variability in Chl-a (up to 2–3 fold) [39] may further reduce the agreement between discrete field measurements and daily satellite products. Despite these limitations, satellite data remain valuable for resolving relative temporal trends and identifying transient bloom dynamics that are otherwise difficult to capture with field observations alone.

The satellite-derived Chl-a record further indicates that the phytoplankton enhancement induced by Typhoon Maria was short-lived. Chl-a returned to near-background levels within approximately one week after the typhoon passage, which is shorter than the bloom development observed after Typhoon Morakot, where enhanced diatom abundance persisted for approximately 10 days after passage [6], and much shorter than the month-long persistence reported in global tropical cyclone analyses [12].

Although phytoplankton taxonomic composition was not directly examined in the present study, previous field observations in the southern East China Sea provide useful regional context for interpreting possible phytoplankton responses to typhoon forcing. Chung et al. [6] reported that after Typhoon Morakot, the micro-phytoplankton assemblage shifted from pre-typhoon dominance by *Trichodesmium* and *Gymnodinium* spp. to a post-typhoon diatom bloom dominated by chain-forming centric diatoms, particularly *Chaetoceros* spp. In addition, satellite- and model-based analyses by Menkes et al. [12] suggested that post-cyclone Chl-a responses may reflect different phytoplankton growth dynamics, with smaller pico-/nanophytoplankton peaking earlier and diatom responses occurring later. These studies suggest that the timing and duration of post-typhoon Chl-a enhancement may depend not only on nutrient supply but also on phytoplankton functional-group responses and grazing pressure. However, because species-level or group-specific phytoplankton data were not available in the present study, we interpret Chl-a changes strictly as variations in phytoplankton biomass rather than taxonomic succession.

This rapid dissipation likely reflects the combined effects of declining nutrient supply and enhanced grazing pressure. Because no strong surface cooling or sustained nutrient outcropping was observed after Typhoon Maria, the additional nutrient input may have weakened rapidly, limiting the maintenance of phytoplankton biomass. In parallel, grazing likely played a key role in accelerating bloom termination. Zooplankton grazing is widely recognized as a major top-down control on phytoplankton biomass, trophic transfer, and carbon export, and recent studies have further shown that grazing dynamics represent a major source of uncertainty in marine carbon-cycle and carbon export estimates [40,41]. In the East China Sea, regional studies also demonstrate that grazing can strongly regulate phytoplankton production and standing stocks; for example, protist grazing can consume a large fraction of picophytoplankton production, particularly during summer, and microzooplankton grazing may balance much of the phytoplankton production in surface waters [42,43]. This top-down control also affects carbon cycling by regulating how phytoplankton-derived carbon is partitioned among respiration, trophic transfer, fecal-pellet production, and export [44]. Previous studies have shown that typhoon-induced diatom blooms can collapse within 24 h under intense copepod grazing, despite prior nutrient enrichment [6]. Such rapid biological removal is consistent with the elevated post-typhoon temperatures (22–28 °C) observed in this study, which favor the growth of herbivorous protists whose grazing rates can exceed phytoplankton growth [45]. Enhanced grazing pressure has also been reported during the same typhoon event [46], further supporting the importance of top-down control in driving the rapid decline in phytoplankton biomass. However, because zooplankton abundance and grazing rates were not directly measured in the present study, grazing should be regarded as a plausible mechanism supported by previous studies rather than direct evidence from our dataset.

It is important to note that typhoon-induced phytoplankton blooms are not ubiquitous. Previous studies have shown that only a fraction of typhoons lead to significant increases in Chl-a [11,12,47], highlighting the complex interplay between physical forcing, nutrient availability, and biological responses. In addition, although nutrient enrichment may stimulate phytoplankton growth, typhoon-induced mixed-layer deepening and, in shallow waters, increased turbidity may reduce light availability; however, bottom-resuspension effects were likely limited in this study because all stations were deeper than 65 m. The results of this study underscore the importance of considering both temporal variability and vertical structure when interpreting phytoplankton responses to typhoon disturbances, as surface observations alone may not fully capture ecosystem-scale changes.

### 4.3. Enhanced Microbial Activity Without Biomass Accumulation

Primary production responses to Typhoon Maria exhibited substantial variability and were not statistically significant, despite apparent increases in magnitude. Both particulate primary production (PPP) and dissolved primary production (DPP) showed large relative increases (75.1 ± 105.2% and 99.5 ± 163.8%, respectively), yet no significant differences were detected between pre- and post-typhoon periods (Table 3; *p*-value > 0.05). This pattern is consistent with the lack of significant changes in surface nutrient and chlorophyll-a concentrations, and likely reflects the combined effects of spatial heterogeneity and the discrete timing of sampling relative to the transient nature of typhoon-induced responses. Nevertheless, a positive relationship between nitracline shoaling and primary production was observed, suggesting that vertical nutrient supply remained an important driver of phytoplankton activity, even if not fully captured by station-based comparisons.

Bacterial dynamics further highlight the complex coupling between physical forcing and microbial processes. Field observations showed that bacterial biomass (BB) remained nearly unchanged (−1.1%), while bacterial production (BP) increased modestly (22.6%) following the typhoon. However, neither response was statistically significant (Table 3; *p*-value > 0.05). In contrast, bacterial specific growth rate (Bμ) increased significantly from 0.15 ± 0.03 to 0.20 ± 0.07 d^−1^ (~35% increase; Table 3; *p*-value < 0.05), indicating enhanced bacterial turnover despite relatively stable biomass.

This pattern suggests that typhoon-induced nutrient and organic matter inputs stimulated bacterial metabolic activity, but did not translate into biomass accumulation. One plausible explanation is that top-down controls, such as grazing, constrained bacterial standing stocks. Enhanced grazing pressure following typhoon events has been reported [46], supporting the interpretation that intensified predation may offset biomass increases even under elevated substrate availability.

Such event-scale responses contrast with findings from seasonal comparisons. Shiah et al. [5] documented substantial increases in bacterial biomass, production, and growth rate during typhoon seasons, indicating strong microbial enhancement under sustained disturbance. The discrepancy suggests that bacterial responses to individual typhoon events are rapid and transient, primarily expressed as increased metabolic rates (e.g., Bμ), whereas prolonged or repeated typhoon forcing may be required to generate measurable increases in biomass. These results indicate that typhoon forcing enhances microbial carbon processing rates without necessarily increasing bacterial standing stocks. This interpretation is consistent with the broader view that marine microbes mediate organic carbon transformation through production, respiration, and remineralization, linking short-term ecological responses to ocean carbon cycling [48].

Bacterial respiration (BR) generally increased following the typhoon, although the change was not statistically significant (*p*-value > 0.05). As a result, bacterial carbon demand (BCD = BP + BR) also showed an increasing trend, indicating enhanced microbial carbon processing. Bacterial growth efficiency (BGE) increased in most stations, suggesting a greater proportion of carbon being allocated to biomass production rather than respiration, although spatial variability remained. Overall, these results indicate that typhoon forcing enhances microbial carbon processing rates without necessarily increasing bacterial standing stocks, highlighting the rapid turnover and redistribution of carbon following episodic disturbance.

## 5. Conclusions

This study demonstrates that the biogeochemical responses to Typhoon Maria are characterized by strong temporal and vertical decoupling, leading to a transient yet dynamically important reorganization of carbon flow in the East China Sea. While surface temperature, nutrients, and chlorophyll-*a* exhibited limited or no significant changes, depth-integrated measurements revealed clear enhancements in nutrient availability and phytoplankton biomass, indicating that typhoon-induced effects were primarily expressed below the surface and may be underestimated by surface observations alone.

Phytoplankton responses were short-lived, with satellite observations showing that blooms dissipated within approximately one week—considerably shorter than previously reported cases. This rapid attenuation reflects the combined effects of limited and short-lived nutrient supply and strong biological controls, particularly grazing. As a result, newly fixed carbon was rapidly redistributed through sinking and trophic transfer rather than retained within the phytoplankton pool.

Microbial responses further reveal that typhoon forcing primarily enhances carbon processing rather than biomass accumulation. Although bacterial biomass and production showed no significant increases, bacterial specific growth rate increased significantly, indicating accelerated microbial turnover. This decoupling between activity and standing stock suggests that bacterial communities rapidly utilized newly available organic substrates, while top-down controls constrained biomass accumulation. In contrast to seasonal-scale observations, where sustained typhoon forcing enhances bacterial biomass, the response to a single event is rapid, transient, and primarily reflected in metabolic rates.

Overall, these findings highlight that typhoon-driven disturbances promote rapid carbon turnover and redistribution through the microbial loop without necessarily increasing biomass stocks. Together, these results suggest that typhoon forcing shifts the ECS ecosystem toward rapid carbon turnover rather than sustained biomass accumulation. This underscores the importance of considering temporal dynamics, vertical structure, and trophic interactions when evaluating ecosystem responses to episodic forcing, and suggests that event-scale processes may play a critical role in regulating carbon cycling in marginal seas.

## Figures and Tables

**Table 1:** Sampling dates relative to the passage of Super Typhoon Maria. Negative and positive values indicate days before and after the typhoon passage, respectively.

Station	Pre-Typhoon	Post-Typhoon
1	−4	+6
1A	−4	+6
2	−2	+3
3	−2	+4
4	−2	+4
5	−2	+4
34	−3	+5
35	−3	+5

**Table 2:** Summary of hydrographic and biogeochemical parameters before and after the passage of Super Typhoon Maria. Values are presented as range and mean ± standard deviation (SD). Statistical significance was assessed using transformed one-sample *t*-tests; n.s. indicates no significant difference (*p* > 0.05). DIN denotes the sum of nitrate, nitrite, and ammonium.

Parameter	Unit	Pre-Typhoon	Post-Typhoon	Difference
Temperature	°C	26.84–28.24 (27.76 ± 0.49)	22.52–28.16 (26.96 ± 1.88)	n.s.
Salinity	psu	33.70–34.18 (34.02 ± 0.15)	33.75–34.48 (34.03 ± 0.22)	n.s.
NO_3_	μM	0.00	0.00–1.88 (0.24 ± 0.67)	n.s.
NO_2_	μM	0.03–0.19 (0.10 ± 0.07)	0.00–0.32 (0.08 ± 0.10)	n.s.
NH_4_	μM	0.00–0.43 (0.07 ± 0.15)	0.00–0.27 (0.14 ± 0.10)	*p* = 0.006
DIN (surface)	μM	0.03–0.46 (0.17 ± 0.13)	0.00–2.07 (0.46 ± 0.66)	n.s.
DIN (water column)	mmol m^−2^	0.02–1.01 (0.31 ± 0.33)	0.04–1.14 (0.41 ± 0.40)	*p* = 0.016
Nitracline	m	28.60–80.00 (52.31 ± 17.74)	0.00–80.00 (43.10 ± 24.37)	n.s.
Chl-a (surface)	mg m^−3^	0.20–0.52 (0.34 ± 0.12)	0.15–1.54 (0.53 ± 0.46)	n.s.
Chl-a (water column)	mg m^−2^	18.92–42.88 (33.49 ± 8.39)	28.98–67.84 (45.63 ± 14.96)	*p* = 0.019

**Table 3:** Summary of microbial parameters before and after the passage of Super Typhoon Maria. Values are presented as range and mean ± standard deviation (SD). Statistical significance was assessed using transformed one-sample *t*-tests; n.s. indicates no significant difference (*p* > 0.05). Abbreviations—PPP, DPP, and TPP: particulate, dissolved, and total primary production, respectively; BB: bacterial biomass; BP: bacterial production; Bμ: bacterial specific growth rate; BR: bacterial respiration; BCD: bacterial carbon demand; BGE: bacterial growth efficiency.

Parameter	Unit	Pre-Typhoon	Post-Typhoon	Difference
PPP	mg C m^−3^ d^−1^	335–790 (584 ± 195)	518–1823 (935 ± 543)	n.s.
DPP	mg C m^−3^ d^−1^	263–654 (504 ± 164)	388–2827 (989 ± 1040)	n.s.
TPP	mg C m^−3^ d^−1^	598–1444 (1088 ± 357)	951–4650 (1923 ± 1570)	n.s.
PER	%	44.0–48.6 (46.3 ± 1.8)	40.8–60.8 (46.3 ± 8.3)	n.s.
BB	g C m^−2^	493–1048 (671 ± 175)	354–1180 (635 ± 268)	n.s.
BP	g C m^−2^ d^−1^	70–114 (95 ± 16)	65–183 (116 ± 36)	n.s.
Bμ	d^−1^	0.10–0.21 (0.15 ± 0.03)	0.12–0.31 (0.20 ± 0.07)	*p* = 0.023
BR	g C m^−2^ d^−1^	2494–4371 (3388 ± 784)	2488–5020 (3833 ± 876)	n.s.
BCD	g C m^−2^ d^−1^	2578–4472 (3483 ± 792)	2552–5203 (3950 ± 908)	n.s.
BGE	%	2.25–4.38 (2.81 ± 0.66)	2.43–3.52 (2.92 ± 0.40)	n.s.

## Data Availability

The datasets generated during the current study will be deposited in the database required by the funding agency (National Science and Technology Council, Taiwan). Before formal deposition, the data are available from the corresponding author upon reasonable request.

## Supplementary Materials

The following supporting information can be downloaded at https://www.mdpi.com/article/10.3390/biology15131007/s1, Table S1: Sampling depths at each station during the pre- and post-typhoon cruises in the East China Sea; Table S2: Summary of phosphate and silicate concentrations before and after the passage of Super Typhoon Maria. Values are presented as ranges and mean ± standard deviation.
