# Supplementary material for: Event-Scale Responses of Phytoplankton and Heterotrophic Bacterial Biomass and Production to Super Typhoon Maria in the East China Sea
Source: Biology (Basel). 2026 Jun 25;15(13):1007. doi: 10.3390/biology15131007 (PMC13359970; doi:10.3390/biology15131007)
Supplement: Supplementary file 1 [file biology-15-01007-s001.zip › biology-4357571-supplementary.pdf]

Table S1. Sampling depths at each station during the pre- and post-typhoon cruises in the East China Sea.

Station	Sampling Depth (m)
1	5, 10, 25, 50, 75, 100, 125, 150, 200
1A	5, 10, 25, 50, 75, 100, 125, 150, 200
2	5, 10, 25, 50, 75, 100
3	5, 10, 20, 30, 50, 70
4	5, 10, 20, 30, 50, 75
5	5, 10, 20, 30, 45, 65
34	5, 10, 25, 50, 75, 100
35	5, 10, 25, 50, 75, 100

Table S2. Summary of phosphate and silicate concentrations before and after the passage of Super Typhoon Maria. Values are presented as ranges and mean ± standard deviation.

Parameter	Unit	Pre-typhoon	Post-typhoon
PO_4	μM	0.04–0.15<br>(0.07 ± 0.04)	0.00–0.15<br>(0.03 ± 0.05)
PO_4<br>(water column)	mmol m^{-2}	0.003–0.07<br>(0.02 ± 0.02)	0.01–0.07<br>(0.03 ± 0.02)
SiO_4	μM	1.89–3.44<br>(2.51 ± 0.46)	1.50–3.61<br>(2.33 ± 0.82)
SiO_4<br>(water column)	mmol m^{-2}	0.16–1.20<br>(0.49 ± 0.34)	0.14–1.32<br>(0.58 ± 0.43)
